# Impact of obesity on outcomes of extracorporeal membrane oxygenation support: a systematic review and meta-analysis

**DOI:** 10.1186/s12890-024-02971-5

**Published:** 2024-03-28

**Authors:** Xinhua Huang, Xiaoqing Lin

**Affiliations:** Department of Geriatric, HuZhou Third Municipal Hospital, the Affiliated Hospital of Huzhou University, 2088 Tiaoxi East Road, Wuxing District, Huzhou City, Zhejiang Province China

**Keywords:** Obesity, Mortality, Extracorporeal membrane oxygenation, Morbidity

## Abstract

**Background:**

Extracorporeal membrane oxygenation (ECMO) is used when standard methods of standard treatment methods are not successful. Obese patients present unique challenges during ECMO due to large body size hindering sufficient flows, difficulties with patient positioning and anatomical landmark identification, and restricted radiology scans. This meta-analysis aims to investigate the impact of obesity on the outcomes of patients undergoing ECMO.

**Methods:**

Databases (PubMed, Embase, and Scopus databases) were searched to identify relevant studies published until July 2023. Data were reported as odds ratios (OR) with 95% confidence interval (CI), and the descriptive data were reported as standard difference of means (SDM) by a random effects model.

**Results:**

A literature search identified 345 studies. Of them, 18 studies met the inclusion criteria. The findings from the meta-analysis revealed no significant association between obesity and survival outcomes after ECMO (odds ratio (OR): 0.91, 95% confidence interval (CI): 0.70–1.17, p: 0.46). Moreover, no comparative significant differences were found between obese and non-obese individuals on the duration of ECMO procedure (standardized mean difference (SMD): 0.07, -0.03–0.17), length of hospital stay (-0.03, -0.19 to 0.12), and duration of ventilation support (-0.10, -0.44 to 0.24).

**Conclusion:**

The meta-analysis findings suggest no significant impact of obesity on the survival outcomes after the ECMO procedure. There was no significant impact of obesity on the duration of ECMO procedures, length of hospital stay, and duration of ventilation support.

**Supplementary Information:**

The online version contains supplementary material available at 10.1186/s12890-024-02971-5.

## Introduction

Obesity is a global health issue that presents formidable challenges to healthcare providers, particularly during life-saving interventions such as extracorporeal membrane oxygenation (ECMO) procedures [1–3]. Obesity, as defined by the Centers for Disease Control and Prevention, is characterized by a Body Mass Index (BMI) of 30.0 or higher. BMI is calculated by dividing a person’s weight in kilograms by the square of their height in meters, serving as a screening tool for evaluating body fatness. The CDC classifies BMI into four categories: underweight (BMI less than 18.5), healthy weight (BMI 18.5 to < 25), overweight (BMI 25.0 to < 30), and obesity (BMI 30.0 or higher) [4]. ECMO is used in patients with severe respiratory or cardiac failure when other conventional treatments fail [1, 5–7]. However, for obese patients, the management of ECMO remains particularly complex due to the unique anatomical and physiological characteristics of this group of patients [8, 9], the presence of various comorbidities, such as diabetes mellitus and hypertension [10, 11], or difficulties in diagnosing and monitoring [3, 8, 10–12]. However, despite these potential issues, obesity has not been identified as a significant risk factor for hospital mortality in patients with acute lung failure and cardiac diseases [3, 10, 11, 13]. Therefore, existing guidelines do not categorize obesity as an absolute contraindication for ECMO support [3, 10, 11]. There is still a lack of comprehensive reviews summarizing the overall evidence in this area [9, 13–16].

This review and meta-analysis aims to summarize all existing data and evaluate the impact of obesity on outcomes, such as mortality rates post-ECMO, the duration of ECMO procedures, lengths of hospital stays, and ventilation requirements, in patients undergoing ECMO. Our study is particularly relevant to the nursing field due to the integral role nurses play in the care of ECMO patients and may contribute to development of evidence-based guidelines, optimizing the management of obese patients on ECMO and potentially improving their survival and recovery rates in critical care settings.

## Methods

The review was performed in adherence to the guidelines of the Preferred Reporting Items for Systematic Reviews and Meta-Analyses (PRISMA) [17]. The paper was registered at PROSPERO, No. CRD42023448406.

### Search strategy

PubMed, Embase, and Scopus databases were searched by using appropriate keywords. We initially compared the complications with ECMO in between obese and non-obese individuals. Search items were as follows: “Obesity,” “Extracorporeal membrane oxygenation,“, “ECMO”, “Body mass index”, “BMI”, “Impact,” “Outcomes,” “Systematic review,” “Meta-analysis,” “ECMO outcomes,” “ECMO complications,” “ECMO survival,” “ECMO mortality,” “Obesity and critical care,” “ECMO and obesity,” “Extracorporeal life support,” “ECMO effectiveness,” “ECMO complications in obese patients.” These terms were combined using the OR operator to ensure comprehensive coverage of relevant literature.

#### Inclusion criteria (as per the PECOS criteria)

1. Population: Obese individuals who underwent ECMO procedure.
2. Exposure: Extracorporeal membrane oxygenation (ECMO) procedures.
3. Comparison: Non-obese who underwent ECMO procedure.
4. Outcome: Mortality, ECMO procedure duration, length of hospital stay, and length of ventilation.
5. Study Design: Randomized controlled trials (RCTs), cohort studies, case-control studies, and observational studies.
6. Language: English.

#### Exclusion criteria

1. Studies are not reporting comparative outcomes between obese and non-obese individuals who underwent ECMO procedures.
2. Non-English languages studies.
3. Incomplete studies, unavailable data, case reports, editorials, commentaries, and letters.

The eligibility of the identified studies was independently assessed by two reviewers. Each study underwent a thorough evaluation based on predetermined criteria to ensure its relevance to the research question. The literature search was also independently conducted by the same two reviewers to minimize bias and improve the accuracy of study selection.

In instances where discrepancies or differences of opinion arose between the reviewers regarding the inclusion or exclusion of a particular study. The outcome of the discussion between the two reviewers was to address any conflicts and facilitate consensus on the final selection of studies. This consensus-based approach among the reviewers ensured a comprehensive and unbiased selection of studies for inclusion in the meta-analysis.

### Quality assessment

To assess the potential bias in the cohort trials included in this study, ROBINS-I tool was used [18]. Two independent reviewers conducted a thorough evaluation of the methodological quality of the included studies. In cases where discrepancies arose, the outcome of the discussion between the two reviewers was to resolve any disagreements and reach a consensus.

### Data extraction

Data were systematically extracted from the selected studies, and included study type, groups involved, sample size, and age of obese and non-obese cohorts, BMIs of the respective groups, type of ECMO procedures, length of hospital stay, duration of ECMO procedure, mortality events, and period of ventilation.

### Statistical analysis

The Meta-analysis version 3.0 was employed for the statistical analysis. Only a random effects model was implemented [19]. The mortality outcomes between obese and non-obese individuals undergoing ECMO procedures was analyzed. Odds ratios (OR) were computed on the basis of the number of events reported in the included studies. The analysis further examined various factors, including the duration of the ECMO procedure, length of hospital stay, and duration of ventilation. For quantifying these changes standard difference of means (SDM) were computed based on the descriptive data provided in the included studies. Heterogeneity among the studies was assessed by I^2^ statistics. I^2^ values between 0 and 25% indicated negligible heterogeneity, 25–75% indicated moderate heterogeneity, and ≥ 75% indicated substantial heterogeneity [20]. Publication bias was assessed by Duval and Tweedy’s trim and fill procedure [21]. Additionally, as we had used both adjusted and unadjusted values for the primary outcome of mortality in our analysis due to paucity of data present in the included studies, we conducted a leave-one-out sensitivity analysis to strengthen the interpretation of our primary outcome. All analyses conducted in this study adhered to a significance level of 5%.

## Results

### Study selection and characteristics

**Fig. 1:**
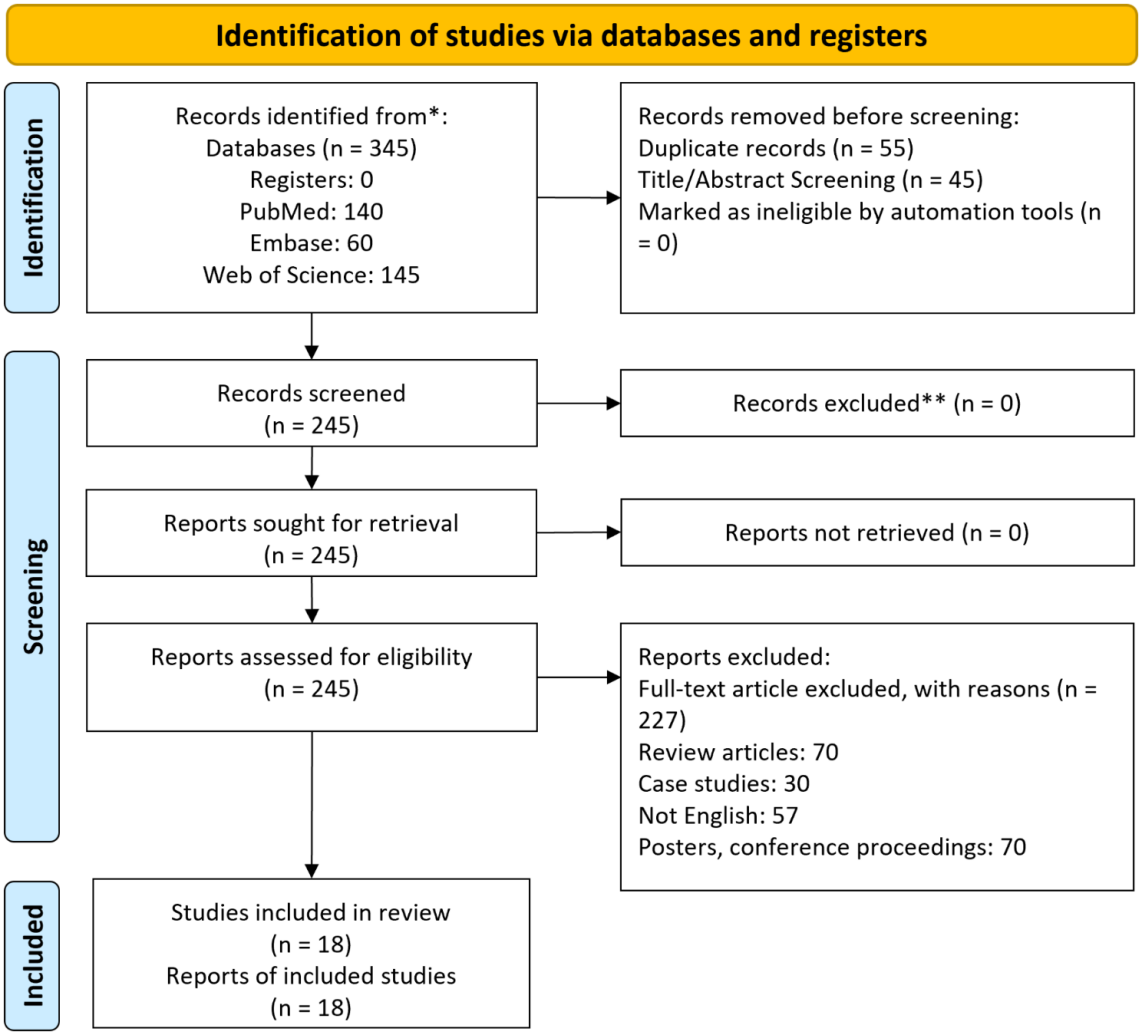
PRISMA flowchart

Among the 345 papers initially retrieved, 18 papers were eligible, as shown in Fig. 1. All of the included studies were retrospective cohort studies [3, 5, 8–11, 14–16, 22–30]. The extracted data from these studies are summarized in Table 1.

**Table 1:** Details of included studies

Study	Design	Country	Groups and sample size	BMI	Age (M ± SD years)	ECMO type	ECMO duration	Mortality	Length of hospital stay (days)	Ventilation time (days)
Prasad, Elkholey et al. (2023)	RCS	USA	N-Obese: 83 (65 M, 19 F) Obese: 126 (92 M, 33 F)	N-Obese: 26.9 ± 2.4 Obese: 34.7 ± 2.6	N-Obese (44.5 ± 11.6) Obese (43.1 ± 10.7)	Venoarterial: 7 Venovenous: 170	N-Obese: 32.4 ± 28.3 days Obese: 31.1 ± 27 days	N-Obese: 42 Obese: 43	–	N-Obese: 39.8 ± 34 Obese: 38.2 ± 32.1
Peetermans, Guler et al. (2023)	RCS	Belgium	N-Obese: 13,822 (9104 M, 4718 F) Obese: 4707 (2724 M, 1983 F)	N-Obese: 26.7 Obese: 42.4	N-Obese (49) Obese (47)	–	N-Obese: 206 h Obese: 237 h	N-Obese: 5608 Obese: 1786	–	–
Javidfar, Zaaqoq et al. (2023)	RCS	USA	N-Obese: 315 (229 M, 86 F) Obese: 39 (21 M, 18 F)	N-Obese: 29 Obese: 44	N-Obese (53) Obese (40)	–	N-Obese: 16 h Obese: 4 h	N-Obese: 126 Obese: 17	N-Obese: 32 Obese: 28	N-Obese: 24 Obese: 16
Lu, Ortoleva et al. (2022)	RCS	USA	N-Obese: 93 (-) Obese: 118 (-)	N-Obese: 25-29.9 Obese: >30	N-Obese (58) Obese (60)	–	N-Obese: 4 days59 Obese: 4 days	N-Obese: 43 Obese: 73	–	–
Djordjevic, Ivanov et al. (2022)	RCS	Germany	N-Obese: 179 (137 M, 49 F) Obese: 59 (43 M, 16 F)	N-Obese: 25 Obese: 33	N-Obese (60) Obese (66)	Central: 59 Peripheral: 179 Concomitant IABP: 171	N-Obese: 76 h Obese: 69 h	N-Obese: 125 Obese: 48	N-Obese: 12 Obese: 10	N-Obese: 8 Obese: 8
Balik, Svobodova et al. (2022)	RCS	Czech Republic	N-Obese: 121 (85 M, 36 F) Obese: 171 (109 M, 62 F)	N-Obese: 26.3 Obese: 35.1	N-Obese (61) Obese (56)	–	N-Obese: 14 days Obese: 14 days	N-Obese: 59 Obese: 85	N-Obese: 27 Obese: 27	–
Powell, Haase et al. (2022)	RCS	USA	N-Obese: 75 (55 M, 20 F) Obese: 30 (19 M, 11 F)	N-Obese: 44.5 Obese: 31.9	N-Obese (45) Obese (37)	–	N-Obese: 838 h Obese: 791.5 h	N-Obese: 28 Obese: 8	N-Obese: 50Obese: 52	–
Mongero, Stammers et al. (2021)	RCS	USA	N-Obese: 129 (102 M, 27 F) Obese: 211 (138 M, 73 F)	N-Obese: ≤30 Obese: >30	N-Obese (50.5) Obese (47.9)	Venoarterial: 24 Venovenous: 304	N-Obese: 552 h Obese: 496 h	–	–	–
Alvarez, O’Malley et al. (2021)	RCS	USA	N-Obese: 59 (42 M, 17 F) Obese: 64 (49 M, 15 F)	N-Obese: 18.5–24.9 Obese: 25-29.9	N-Obese (47) Obese (55)	–	N-Obese: 9 days Obese: 9 days	N-Obese: 24 Obese: 27	–	–
Salna, Fried et al. (2021)	RCS	USA	N-Obese: 111 (80 M, 31 F) Obese: 150 (111 M, 39 F)	N-Obese: 22.4 Obese: 27.3	N-Obese (61) Obese (61)	–	N-Obese: 3.7 days Obese: 4.6 days	N-Obese: 56 Obese: 80	N-Obese: 30 Obese: 26	–
Verkerk, Dzierba et al. (2020)	RCS	USA	N-Obese: 43 (16 M, 27 F) Obese: 38 (11 M, 27 F)	N-Obese: 26 Obese: 37	N-Obese (44) Obese (43)	–	N-Obese: 8.6 days Obese: 11.1 days	N-Obese: 13 Obese: 8	N-Obese: 29.8 Obese: 35.5	–
Merritt-Genore, Lyden et al. (2020)	RCS	USA	N-Obese: 76 (-) Obese: 59 (-)	N-Obese: - Obese: -	N-Obese: - Obese: -	–	N-Obese: 25 Obese: 30	N-Obese: 48 Obese: 49	N-Obese: 24.5 Obese: 20	–
Lee, Moon et al. (2020)	RCS	South Korea	N-Obese: 27 (16 M, 11 F) Obese: 33 (26 M, 7 F)	N-Obese: 21 ± 1.4 Obese: 25.6 ± 2	N-Obese (73.6 ± 10) Obese (66.1 ± 11.3)	–	N-Obese: 7 days Obese: 7 days	N-Obese: 24 Obese: 19	N-Obese: 9 Obese: 14	–
Galvagno, Pelekhaty et al. (2020)	RCS	USA	N-Obese: 37 (-) Obese: 42 (-)	N-Obese: <30 Obese: ≥ 30	N-Obese (28) Obese (39)	–	N-Obese: 15 days Obese: 23.8 days	N-Obese: 10 Obese: 8	–	–
Salna, Chicotka et al. (2018)	RCS	USA	N-Obese: 131 (74 M, 57 F) Obese: 63 (41 M, 22 F)	N-Obese: 25 Obese: 34	N-Obese (47) Obese (41)	–	–	N-Obese: 44 Obese: 28	N-Obese: 27 Obese: 21	–
Cho, Oh et al. (2018)	RCS	South Korea	N-Obese: 58 (41 M, 17 F) Obese: 26 (19 M, 7 F)	N-Obese: 21.4 ± 2.5 Obese: 27.8 ± 2.5	N-Obese (58.1 ± 14.7) Obese (52.3 ± 14.8)	Venoarterial: 8 Venovenous: 71 Venoarterial-venous: 5	N-Obese: 17.2 ± 16.4 days Obese: 14 ± 11.6 days	N-Obese: 37 Obese: 8	N-Obese: 58.6 ± 55.1 Obese: 56.1 ± 44.6	–
Lazzeri, Bonizzoli et al. (2017)	RCS	Italy	N-Obese: 45 (-) Obese: 25 (-)	N-Obese: 21.9 ± 2.8 Obese: 33.8 ± 3.1	N-Obese (49.8 ± 15.8) Obese (53.6 ± 11.8)	–	N-Obese: 13.4 ± 13.5 days Obese: 19.2 ± 20.4 days	N-Obese: 28 Obese: 7	–	N-Obese: 19.1 ± 21.4 Obese: 27.2 ± 27.4
Kon, Dahi et al. (2015)	RCS	USA	N-Obese: 43 (29 M, 14 F) Obese: 12 (2 M, 10 F)	N-Obese: 29 Obese: 49	N-Obese (35) Obese (43.5)	–	N-Obese: 9 days Obese: 14 days	N-Obese: 18 Obese: 4	N-Obese: 28 Obese: 35	–

ECMO: Extracorporeal membrane oxygenation, IABP: Intra-aortic balloon pump, N-Obese: Non-obese, RCS: Retrospective cohort study

### Participant information

The analysis incorporated data from 21,361 patients undergoing ECMO. A total of 15,447 patients (10,075 males, 5129 females) were non-obese, and 5914 (3405 males, 2323 females) were obese. Sex distribution was not reported by four of the included studies [5, 15, 16, 24]. The mean age of the non-obese and obsess patients was 51.3 ± 10.6 years and 50.1 ± 9.4 years, respectively.

### Assessment of study quality

The methodological quality of the cohort studies included in the analysis was evaluated using the ROBINS-I tool [18]. As summarized in Table 2, there was a high risk of bias across the included studies. However, it was noted that several studies had missing data, showed signs of deviation from intervention, and selection bias suggesting possible sources of bias (Table 2).

**Table 2:** Risk of bias as per the ROBINS-I methodological tool (low risk of bias: +, high risk of bias: -, lack of clarity:?)

Study	Confounding bias	Selection bias	Deviation from the intervention	Missing data	Measurement of outcomes	Selective reporting	Classification of the intervention	Overall risk of Bias
Prasad, Elkholey et al. (2023)	+	+	?	+	+	?	+	Moderate
Peetermans, Guler et al. (2023)	+	+	+	−	+	+	+	Moderate
Javidfar, Zaaqoq et al. (2023)	+	+	+	+	+	+	+	Low
Lu, Ortoleva et al. (2022)	+	?	?	+	+	+	+	Moderate
Djordjevic, Ivanov et al. (2022)	+	+	+	−	+	+	+	Moderate
Balik, Svobodova et al. (2022)	+	+	+	+	+	?	+	Moderate
Powell, Haase et al. (2022)	+	+	+	−	+	+	+	Moderate
Mongero, Stammers et al. (2021)	+	+	?	?	+	?	+	Serious
Alvarez, O’Malley et al. (2021)	+	+	+	−	+	+	+	Moderate
Salna, Fried et al. (2021)	+	+	?	+	?	+	+	Moderate
Verkerk, Dzierba et al. (2020)	+	+	+	+	+	+	+	Low
Merritt-Genore, Lyden et al. (2020)	+	?	+	?	+	+	+	Moderate
Lee, Moon et al. (2020)	+	+	+	−	+	+	+	Moderate
Galvagno, Pelekhaty et al. (2020)	+	+	?	?	+	?	+	Serious
Salna, Chicotka et al. (2018)	+	+	+	−	+	+	+	Moderate
Cho, Oh et al. (2018)	+	+	+	−	+	+	+	Moderate
Lazzeri, Bonizzoli et al. (2017)	+	+	?	?	+	?	+	Serious
Kon, Dahi et al. (2015)	+	+	+	−	+	+	+	Moderate

### Mortality

**Fig. 2:**
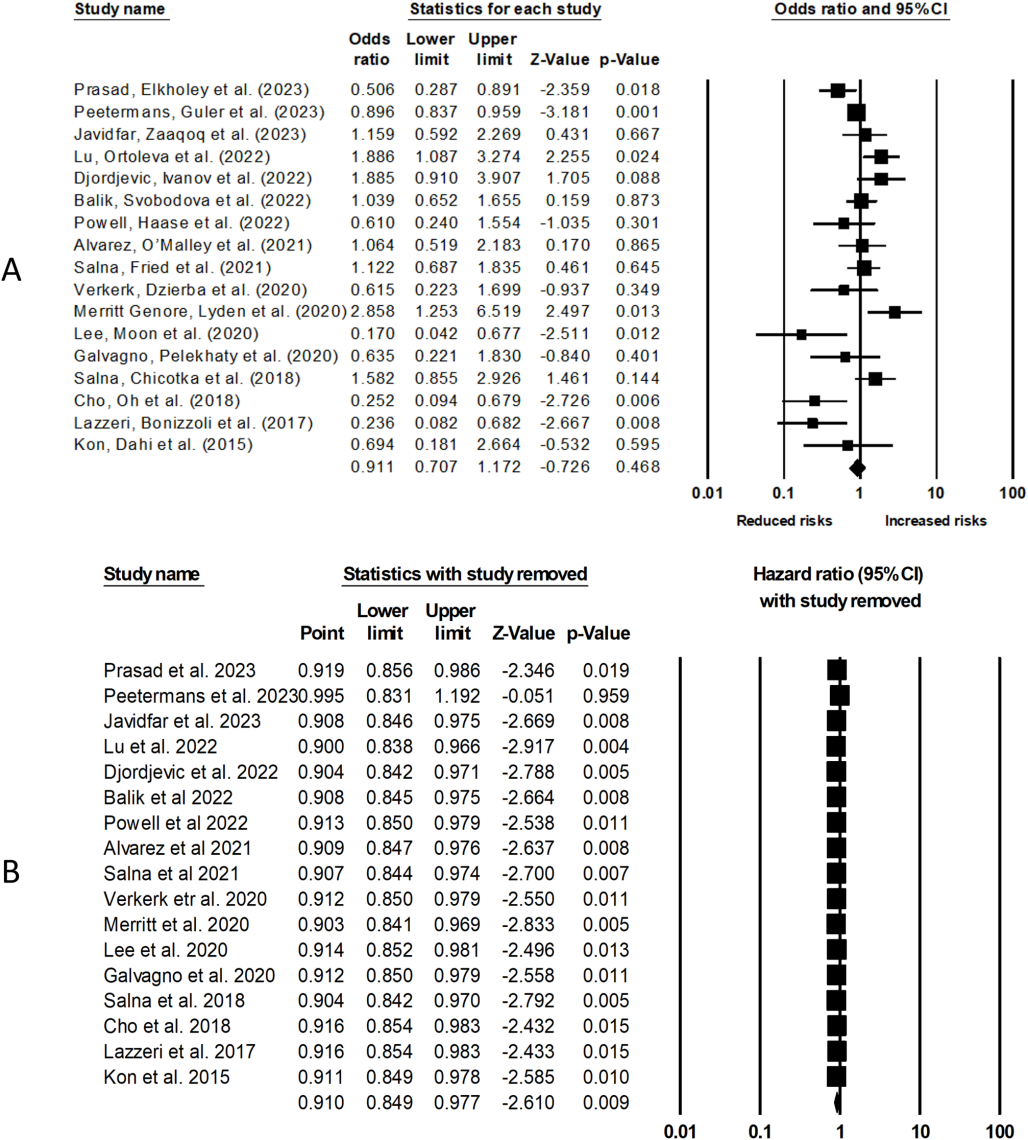
(**A**) The comparative outcome between obese and non-obese patients who underwent ECMO procedure on overall mortality. (**B**) Leave one out sensitivity analysis

Our analysis of 17 cohort studies (Fig. 2A) indicates a non-significant effect of obesity on overall mortality in patients who underwent ECMO procedure (odds ratio [OR]: 0.91, 95% confidence interval [CI]: 0.70–1.17, *p* = 0.46), with moderate heterogeneity (I^2^: 38.5%). Figure 2B indicates leave one out sensitivity analysis for this analysis.

### Publication bias

**Fig. 3:**
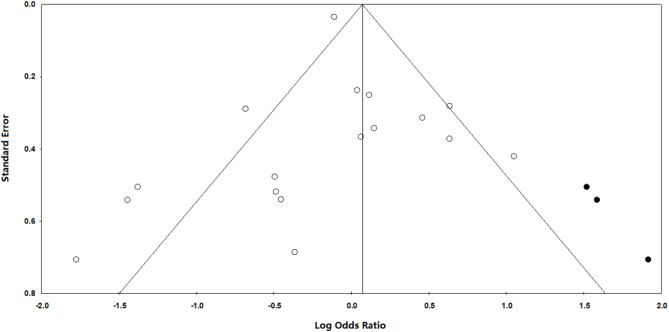
The trim and fill method by Duval and Tweedy provides a visualization of how publication bias can impact study results

To assess publication bias, we employed Duval and Tweedy’s trim and fill method, which estimates the number of missing studies on either side of the mean effect of the funnel plot. According to this method, three studies were missing on the right side of the mean effect. The overall random effect models provided a point estimate and 95% confidence interval for the studies as 0.90 (95% CI 0.85–0.96). The trim and fill imputed point estimates were calculated as 0.92 (95% CI 0.86–0.98). The results of the publication bias assessment are presented in Fig. 3.

### ECMO procedure duration

**Fig. 4:**
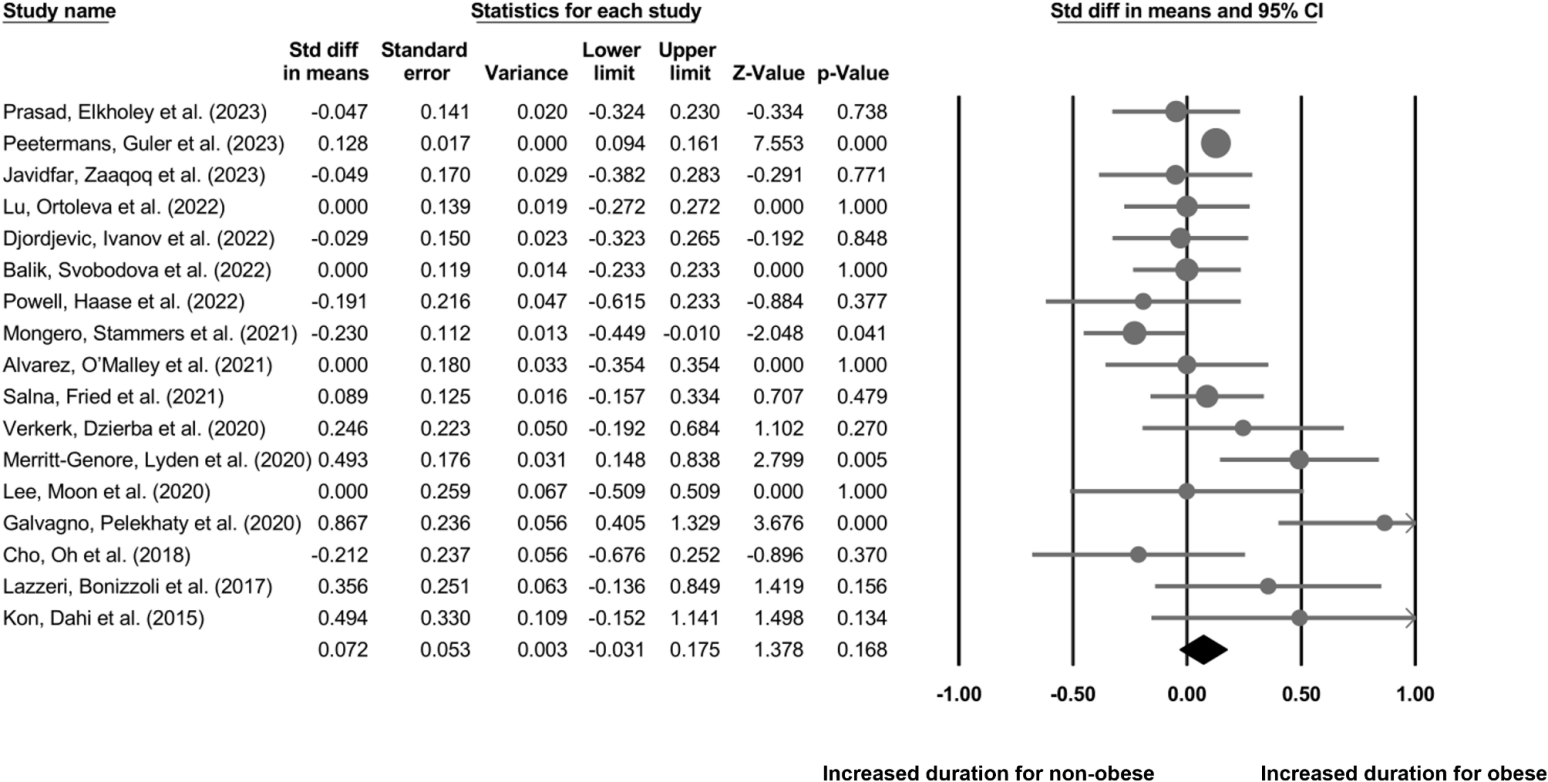
The comparative outcome between obese and non-obese individuals who underwent ECMO procedure on the overall duration of the procedure

Our analysis of 18 cohort studies (Fig. 4) indicates no difference in the duration of ECMO procedure in obese individuals as compared to non-obese individuals (Standardized difference in means: 0.07, 95% CI: -0.03–0.17, *p* = 0.16) with negligible heterogeneity (I^2^: 24.5%).

### Length of hospital stay

**Fig. 5:**
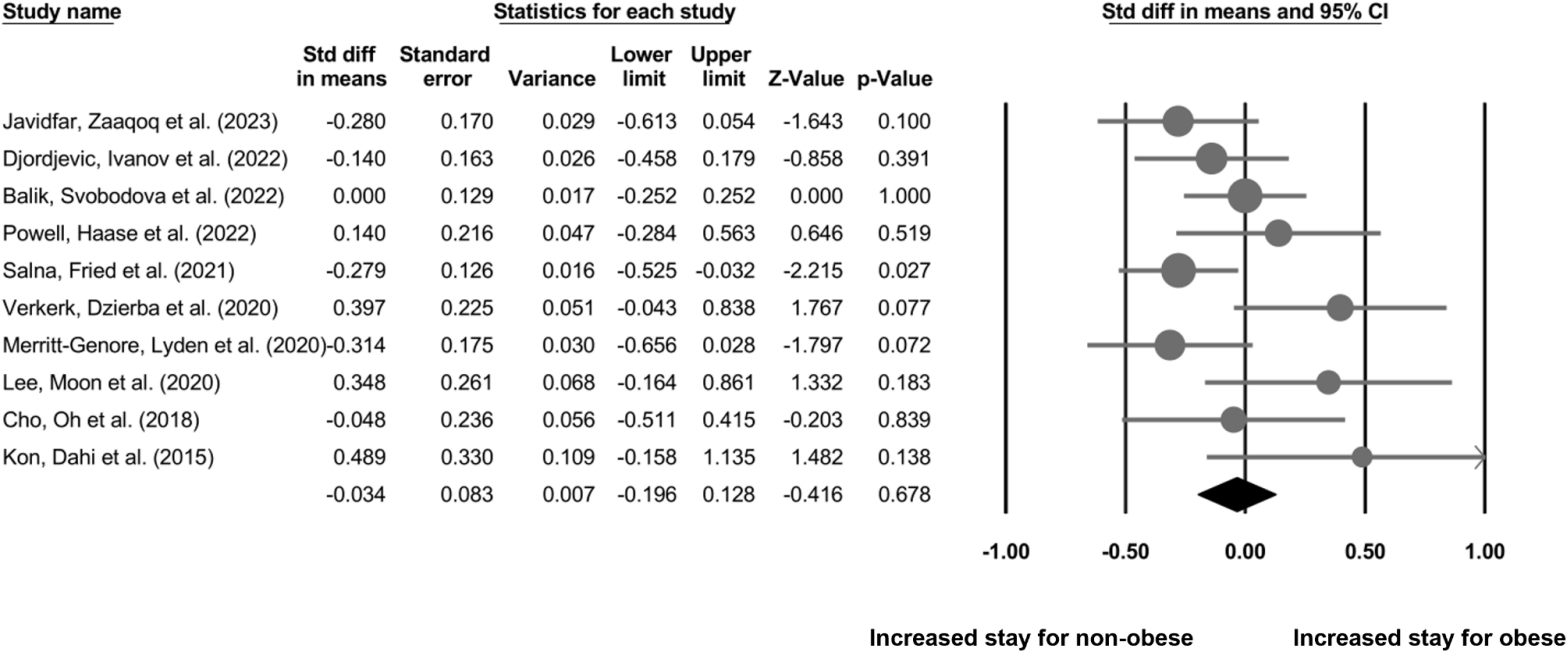
The comparative outcome between obese and non-obese individuals who underwent ECMO procedure on the length of hospital stay

Our analysis of 11 cohort studies (Fig. 5) indicates a non-significant difference in the length of hospital stay for non-obese individuals as compared to the obese individuals who underwent ECMO procedure (SDM: -0.03, 95% CI: -0.19–0.12, *p* = 0.67) with negligible heterogeneity (I^2^: 8.8%).

### Duration of ventilation

**Fig. 6:**
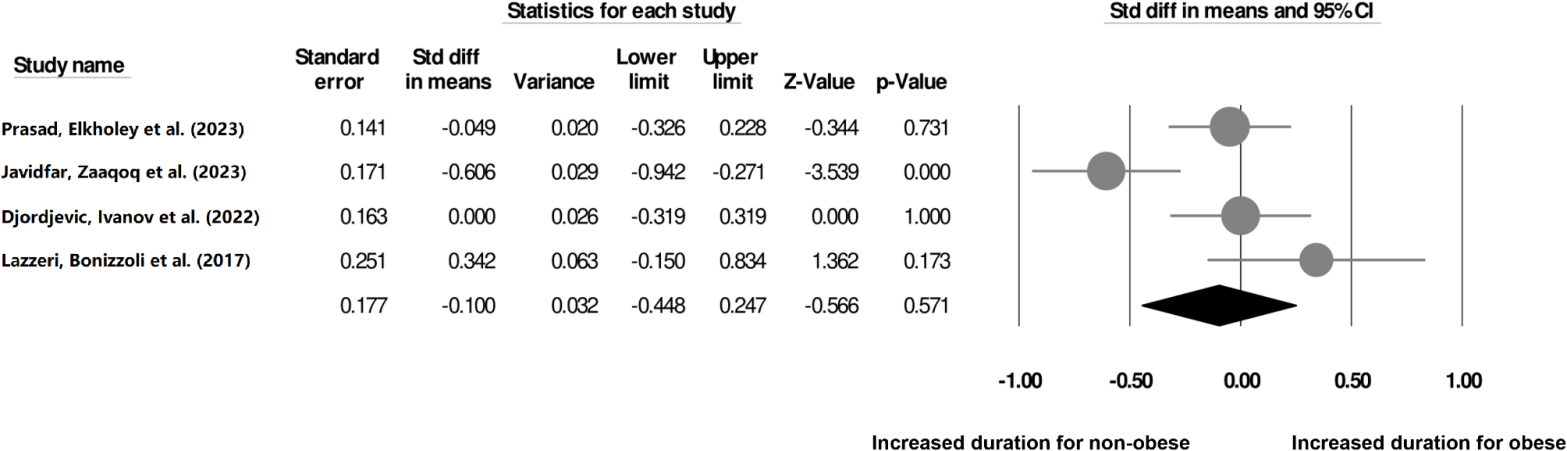
The comparative outcome between obese and non-obese individuals who underwent ECMO procedures during the period of ventilation

Our analysis of 4 cohort studies (Fig. 6) indicates a non-significant difference in the duration of ventilation for non-obese individuals as compared to the obese individuals who underwent ECMO procedure (SDM: -0.10, 95% CI: -0.44–0.24, *p* = 0.57) with negligible heterogeneity (I^2^: 12.9%).

## Discussion

The results of our meta-analysis showed no substantial correlation between obesity and post-ECMO mortality outcomes. No difference was found between obese and non-obese patients in terms of ECMO procedure duration, the length of hospital stay, and the duration of ventilation required. Our results are particulary important for nursing professionals who are providing holistic care during ECMO interventions, and may serve to inform nursing practices to tailor their care strategies effectively.

Obesity may lead to complications during ECMO procedures through several possible mechanisms [19, 20, 23]. The increased adipose tissue in obese patients can pose challenges during cannulation, as it obscures the underlying vessels and makes it difficult to identify suitable cannulation sites [1, 3, 6–9, 12, 19]. This may result in suboptimal cannula positioning or vascular injuries during insertion, leading to hemorrhage or improper ECMO flow [23, 29, 31]. Altered respiratory mechanics in obese patients, characterized by reduced lung compliance and increased airway resistance, can impact efficient oxygenation and carbon dioxide removal by the ECMO system [23, 29]. As a result, inadequate oxygenation and perfusion can cause hypoxemia and inadequate organ support [5]. For instance, in obese individuals, changes in chest wall resistance and lung compliance can complicate respiratory function [32]. With respect to ECMO, oxygenation challenges may arise due to ventilation-perfusion mismatch and atelectasis [33]. The removal of CO2 is impeded by these altered mechanics, potentially resulting in hypercapnia. While ECMO provides extracorporeal support for both oxygenation and CO2 removal, its efficacy is influenced by various external factors, including pump speed, blood flow rate, and the efficiency of the oxygenator and sweep gas [34]. It is essential to recognize that the effectiveness of ECMO is not solely determined by the inherent nature of the lung disease; rather, it is a complex interplay of external parameters. In situations where lung mechanics are altered, patients may require a prolonged duration of ECMO support, allowing for sufficient time for the lungs to recover to a point where they can adequately provide oxygenation and ventilation. Therefore, tailoring ECMO configurations and closely monitoring patients for dynamic adjustments are crucial aspects for optimizing outcomes in these cases.Furthermore, obese patients with pre-existing cardiac issues may experience additional strain on the heart due to the hemodynamic load imposed by the ECMO circuit [5, 14]. This can lead to myocardial ischemia, arrhythmias, or cardiac arrest [17, 18]. Moreover, obesity increases the risk of thromboembolic events, making obese patients more susceptible to clot formation within the ECMO circuit or embolization of clots to vital organs [6, 8, 13, 21]. Impaired immune function in obese patients also heightens the risk of potentially life-threatening ECMO-related infections [3, 9, 10, 12].

In our study, we evaluated the comparative mortality outcomes between obese and non-obese patients, undergoing ECMO. A study by Lu, Ortoleva et al. (2022) [16] also reported lack of association between obesity and mortality in their cohort, and provided several plausible explanations for that observation. Firstly, 944 patients in their cohort had missing BMI data, and the reported in-hospital mortality rate was high (89.1%). Secondly, while comorbidities were considered in the analysis, the severity of illness before cannulation for VA-ECMO was not accounted for. Thirdly, sample size in Lu, Ortoleva et al. (2022) study was small [16], which might have hindered the detection of existing differences. Interestingly Djordjevic, Ivanov et al. (2022) and Merritt-Genore, Lyden et al. (2020) reported reduced mortality outcomes in their obese cohort as compared to the non-obese cohort who underwent ECMO procedure [15, 29]. Here, the authors suggested that perhaps the reduction in the mortality outcomes could be due to the obesity paradox in individuals undergoing ECMO due to the potential benefits provided by the increased adipose tissue. Obese individuals may have greater nutritional and metabolic reserves, which could help them withstand the stress of ECMO support better [35]. Additionally, adipose tissue produces certain protective hormones that may have favorable effects on cardiovascular function and immune response during critical illness [36, 37].

We also did not observe any significant difference in ECMO procedure duration between obese and non-obese individuals. This lack of difference can be explained by recent advancements in ECMO technology and expertise in managing obese patients which minimized any potential procedural delays related specifically to obesity. Additionally, the ECMO circuit could have been adapted to accommodate the larger body size of obese patients, allowing for adequate flow and gas exchange, which could also reduce the differences in procedure duration. Finally, healthcare providers could have developed standardized protocols and tailored strategies for obese patients, optimizing patient positioning and cannula placement, leading to comparable procedure durations. All these reasons can also explain the lack of differences in the duration of hospitalization and ventilation support between obese and non-obese individuals observed in our meta-analysis. Besides, as our findings indicate that obesity does not significantly impact ECMO outcomes. These findings can empower nurses to tailor care strategies for obese patients, address specific challenges, and contribute to collaborative decision-making. Based on this information, nurses can focus on meticulous monitoring, educate and support patients based on evidence, and actively participate in refining protocols to optimize care for obese individuals on ECMO.

## Limitation

Our study, despite its rigorous methodology, is not immune to certain limitations inherent in non-randomized observational studies. Firstly, confounding bias, a common concern in such research designs, arises from the potential influence of unmeasured or unaccounted variables on the observed associations. Although we employed careful study selection criteria and adjusted our analyses where feasible, the diversity in methodologies, patient characteristics, and reporting practices across the included studies introduces the possibility of residual confounding. Addressing confounding bias in the context of our research question is complex. While some studies may have adjusted for relevant factors, the variability in the control of confounders across the literature is a notable limitation. Secondly, there was variation in the reported parameters across the studies included in the review. For instance, mortality and ECMO procedure duration were the most widely evaluated outcomes (i.e., reported by 17 and 18 studies), whereas the duration of ventilation support was only reported by four studies. This variation in unreported data could be a source of heterogeneity in our analyses and could also introduce bias in our results, making generalizability of our findings difficult. Furthermore, while some studies in the literature have employed propensity score matching to address selection bias and confounding, our decision to utilize unadjusted values was pragmatic. The diverse methodologies and variable reporting across studies made it challenging to uniformly apply propensity score matching. This limitation underscores the importance of interpreting our results with caution, recognizing the potential impact of unmeasured confounders. Therefore, we strongly recommend future studies to replicate our findings in large-scale trials with consistent data reporting for obese and non-obese individuals undergoing ECMO procedures to help develop more robust evidence to guide clinicians in selecting appropriate strategies to improve outcomes following ECMO.

## Conclusions

In conclusion, this meta-analysis provides valuable insights into the impact of obesity on ECMO outcomes. It indicates that obesity alone does not significantly affect mortality outcomes or the duration of the ECMO procedure. However, healthcare providers should be aware of the challenges that obese patients may face during ECMO and consider tailored management approaches to optimize their care. This study contributes to a better understanding of the role of obesity in ECMO and can aid in improving the clinical decision-making process for this specific patient population.

### Electronic supplementary material

Below is the link to the electronic supplementary material.

Supplementary Material 1

## Data Availability

Data is provided within the manuscript or supplementary information files.
